# Immune Gene Expression in *Bombus terrestris*: Signatures of Infection Despite Strong Variation among Populations, Colonies, and Sister Workers

**DOI:** 10.1371/journal.pone.0068181

**Published:** 2013-07-15

**Authors:** Franziska S. Brunner, Paul Schmid-Hempel, Seth M. Barribeau

**Affiliations:** Experimental Ecology, Institute of Integrative Biology, ETH Zürich, Zürich, Switzerland; University of Sussex, United Kingdom

## Abstract

Ecological immunology relies on variation in resistance to parasites. Colonies of the bumblebee *Bombus terrestris* vary in their susceptibility to the trypanosome gut parasite *Crithidia bombi,* which reduces colony fitness. To understand the possible origin of this variation in resistance we assayed the expression of 28 immunologically important genes in foraging workers. We deliberately included natural variation of the host “environment” by using bees from colonies collected in two locations and sampling active foraging workers that were not age controlled. Immune gene expression patterns in response to *C. bombi* showed remarkable variability even among genetically similar sisters. Nevertheless, expression varied with parasite exposure, among colonies and, perhaps surprisingly, strongly among populations (collection sites). While only the antimicrobial peptide abaecin is universally up regulated upon exposure, linear discriminant analysis suggests that the overall exposure effect is driven by a combination of several immune pathways and further immune functions such as ROS regulation. Also, the differences among colonies in their immune gene expression profiles provide clues to the mechanistic basis of well-known inter-colony variation in susceptibility to this parasite. Our results show that transcriptional responses to parasite exposure can be detected in ecologically heterogeneous groups despite strong background noise.

## Introduction

The outcome of host-parasite interactions is highly variable. This is because a successful infection results from complex interactions of both host and parasite genotypes and the molecular mechanisms coded by these genes, which are additionally influenced by biotic and abiotic ecological forces. The molecular mechanisms underlying host resistance to specific parasites are much better understood in vertebrates (e.g. the MHC-locus [1], [2]) than invertebrates. However, a few notable immunological elements have been identified in invertebrates that might be involved in specificity of resistance. For example, alternative splicing of the Dscam transcripts (a gene involved in phagocytosis) can produce spectacular isoform diversity in Dipterans [3]. Similarly, the ALP1 gene in *Anopheles gambiae*, which is important for the mosquito’s response to the parasite *Plasmodium falciparum* shows evidence of rapid evolution and high polymorphism [4]. These two genes offer intriguing hints to the mechanistic causes of variation in invertebrate host immune defense, but their generality in other species remains to be seen. Variation in infection outcome could also be a result of differences in either constitutive or induced expression of genes, even when coding sequences are relatively monomorphic. The ecological background of specific host-parasite interactions can dramatically influence the outcome [5] and presumably may also affect immune gene activity of the host. To investigate this idea, we explored the variation in immunological gene expression of bees from different locations in response to infection.

The buff-tailed bumblebee *Bombus terrestris* L. and its gut parasite *Crithidia bombi* [6] are a model of host-parasite interactions and the resulting co-evolutionary processes [7]. *B. terrestris* is a common and important European pollinator. It lives in colonies headed by a single queen, which mates only once, producing a genetically homogeneous group of sister workers. *C. bombi* is a trypanosome gut parasite of bumblebees that is transmitted via ingestion of parasite cells spread in feces by other infected individuals [8]. *C. bombi* reduces worker lifespan under harsh conditions but is otherwise avirulent in workers [9]. Infection does, however, strongly reduce female fitness by reducing colony establishment success of young queens in the next generation [9]. Parasite prevalence under natural conditions is high [10]; hence, the general effect of *C. bombi* on the populations of its hosts is likely to be important. Specific clonal isolates of *C. bombi* differ in their infection ability towards different host genotypes [11]. These specific interactions are also moderated by environmental factors [12]. Therefore, an investigation of the molecular basis of interactions between *B. terrestris* and *C. bombi* should take into account both ecological context and genetic identity of hosts and parasites.

The insect immune system lacks the lymphocytes of vertebrates but can nevertheless be very specific in its response to different challenges [13]. The system contains four main interconnected immune pathways: the Toll and the Imd pathways (called NF-κB pathways because they involve nuclear factor -κB -like transcription factors); the JNK pathway, which shares recognition molecules with the Imd pathway; and the JAK/STAT pathway. Typical responses of the insect immune system include the secretion of antimicrobial peptides, hemocyte activity (encapsulation or phagocytosis), melanization reactions, and coagulation [14].

So far, the expression of only a few genes has been assessed in bumblebees infected with *C. bombi* (*c.f.* Table 1). Riddell et al. [15] found that colonies respond differently to different parasite strains such that the expression of the antimicrobial peptide genes varied in relation to which parasite strain infected which host colony. The most recent study on immune expression of *B. terrestris* in response to *C. bombi*, using an SSH library, found that immune pathway signaling genes are most prominently up regulated upon exposure to *C. bombi* [16]. Based on this previous work, we now much more broadly surveyed the expression of genes involved in the constitutive and induced response when *B. terrestris* is infected by the trypanosome gut parasite *C. bombi.* We were particularly interested in the standing and induced immunological variation within colonies as this variation could be important in preventing the spread of parasites and should reflect natural expression levels as far as possible. Bumblebee colonies are genetically homogeneous because of their haplodiplod genetic system and monogamous mating. Artificially increasing genetic diversity in *B. terrestris* colonies by inseminating queens with sperm from multiple males produced more resistant colonies [17]. Similarly, variation in immune response within colonies may be adaptive to the colony as a whole. To capture this variation we specifically targeted foraging workers. Foragers are the individuals that are most likely to encounter parasites in the environment and bring them back to the colony. Focusing on these workers that are the ‘front line’ in parasite encounters we assess how a colony deals with infection at first encounter. Furthermore, we want to capture a “snapshot” of the immune response across a colony rather than an age specific response to parasite exposure. *B. terrestris* workers and colonies change in their immunological profiles as they age [18]. Such a snapshot may reflect the naturally occurring selective situation better than the fully controlled experiment, since it describes the effect of an infection against the naturally varying background.

**Table 1:** Synopsis of immune gene regulation effects found by previous studies in comparison with our results.

Gene	Effects found in previous study	Effects found in our study	Differences in study designwith respect to our study
hemomucin (pathogenrecognition molecule)	Up regulation upon *Crithidia*infection [22]	No significant infection effect	Different time point for gene expression assessment (10 days vs 18 hrs post infection), workers age controlled
relish (signaling molecule,Imd pathway)	Tendency for up regulation upon*Crithidia* infection [22]	No significant infection effectbut differently expressedbetween collection sites	
basket (signaling molecule,JNK pathway)	Down regulation upon bacterialchallenge [21]	No significant infection effect	Responses to wounding and bacterial challenge tested, commercial bumblebee colonies used
TEP A (effector of theJAK/STAT pathway)	Down regulated upon wounding [21]	No significant infection effect	
abaecin (AMP)	Up regulation upon wounding [21]	Significant up regulation upon infection	
	Up regulation 12 hours after infection,strong variation among individuals [16]		Only one colony considered, workers age controlled, commercial bumblebee colonies used
defensin (AMP)	Up regulation 12 hours after infection,strong variation among individuals [16]	No significant infection effect	
	Up regulation upon wounding, furtherup regulation when including bacterial challenge [21]		Responses to wounding and bacterial challenge tested, commercial bumblebee colonies used
	GxG interaction of host and parasite genotypes on expression levels [15]		commercial bumblebee colonies used, effect of infection on expression levels across colonies not described
	Expression levels dependent on social environment (up regulation in groupliving bees)		commercial bumblebee colonies used, no infection responses tested
hymenoptaecin (AMP)	GxG interaction of host and parasite genotypes on expression levels [15]	No significant infection effect	commercial bumblebee colonies used, effect of infection on expression levels across colonies not described
	Up regulation upon wounding, furtherup regulation when including bacterial challenge [21]		Responses to wounding and bacterial challenge tested, commercial bumblebee colonies used
	strong variation among individuals [16]		Only one colony considered, workers age controlled, commercial bumblebee colonies used
	Expression levels dependent on social environment (up regulation in groupliving bees) [43]		commercial bumblebee colonies used, no infection responses tested
lysozyme(bacteriolytic effector)	Expression levels dependent on socialenvironment (down regulation in beeskept solitary) [43]	Up regulation upon infectionin Neunforn bees	
peroxidase(ROS regulation enzyme)	Up regulation 1–4 hours afterinfection [16]	No significant infection effectbut differences in expressionbetween sites for several ROSregulation enzymes	Only one colony considered, workers age controlled, commercial bumblebee colonies used
transferrin (iron transportationmolecule)	Up regulation after injection with PBS, bacterial challenge and iron overload,peak at 6 hours post treatment [44]	No significant infection effect	*B. ignitus* used as study system, expression levels assessed with Northern blots, only one pool of three workers assessed
ferritin (iron transportationmolecule)	Up regulation after injection with PBS, bacterial challenge and iron overload,peak at 18 hours post treatment [44]	No significant infection effect	
serpin27a (PPO cascadeenzyme)	Expression levels dependent on social environment (up regulation in groupliving bees) [43]	Up regulation upon infectionin Neunforn bees	commercial bumblebee colonies used, no infection responses tested

Colony effects are excluded from the synopsis as they occur in the vast majority of genes.

We also measured immunological gene expression of bees from two locations that differ in parasite prevalence to assess possible geographical variation in immune gene expression. Our main motivation was to explore the standing variation in a typical set of genes associated with immune defence, to investigate how they change upon exposure, and whether there are recognizable statistical patterns in these expression profiles. We were particularly interested in the standing and induced immunological variation within colonies as this variation could be important in preventing the spread of parasites and should reflect natural expression levels as far as possible. The ability to detect responses to exposure through the biological noise of varied ages that exist in a bumblebee colony is a valuable tool as these patterns would be at the core of any immunological-ecological analysis that extends beyond the usual one-case-in-the-laboratory study and would link back to the situation in the wild.

## Materials and Methods

### Bee Collection and Exposure to Crithidia

We collected *Bombus terrestris* queens from two locations in Northern Switzerland (Aesch BL and Neunforn TG) in spring 2011 and allowed them to establish colonies in the lab. No collection permits are needed to collect *B. terrestris* on private land in Switzerland. We received permission from private landowners to collect on their properties. The two collection sites are known to differ in parasite prevalence, and indications for local coadaptation in the *Bombus-Crithidia* system have been found in previous studies [7]. Over the years 2007 to 2011, on average 5.051% of spring queens collected in Neunforn and 12.52% of spring queens collected in Aesch were infected with *C. bombi* (N between 125 and 393 per year and site, Table S1 in File S1). Upon arrival in the lab, feces from the queens were checked for *Crithidia* infections and only colonies from non-infected queens were used in our experiments.

Colony enclosures consisted of a central colony chamber and an outbox where sugar water was provided *ad libitum*. Pollen was fed *ad libitum* within the central colony chamber. Bees could freely move between these two compartments. We took 12 workers from each of the outboxes of 8 colonies (4 from each collection location), not controlled for age, and considered them representative foragers of a colony. These bees were starved for 2 hours and 6 workers from each group were infected by feeding them 10,000 cells of *C. bombi* in 10 µL of 50% sugar water. Mixtures of equal numbers of four different clonal lines of *C. bombi* were used as an infective dose to elicit a broad immune response in the bumblebee host to this parasite (instead of a specific response to one *Crithidia* strain only). We fed the other 6 bees from each colony a sham inoculum, i.e. sugar water without *C. bombi* cells as a control. No bees failed to eat the inoculum. The parasite exposure order was randomized and identical for every colony. 18 hours after exposure or control treatment, we snap-froze all bees in liquid nitrogen and stored them at −80°C until use. This time point was chosen based on previous studies identifying gene expression up regulation for antimicrobial peptides [16] and high PO activity and antibacterial activity [19] around 18 hours post *Crithidia* exposure.

The four *Crithidia* strains used in this experiment were isolated from two spring queens collected in 2008 (one from Neunforn, one from Aesch) and one spring queen each from 2009 and 2010 (both from Aesch). Each strain originated from a single infective cell and was cultured in liquid medium at 27°C and 3% CO_2_ after isolation and then cryogenically stored until they were cultured again immediately before use in exposures [20].

### Genetic Analyses

We dissected bee abdomens and disrupted them with 0.5 g Zirkonium beads at −4 to −10°C using an Omni Bead Ruptor 24 Homogenizer (OMNI International). We then extracted RNA using the RNeasy Plus Mini kit (Qiagen) in 8 randomized extraction groups of 10 to 12 samples each. We checked two to three samples from each extraction group on a 2100 Bioanalyzer (Agilent Technologies) with the RNA 6000 Nano Kit to confirm RNA integrity. When RNA profiles indicated degraded samples, we checked all samples from the same extraction group on the Bioanalyzer and excluded degraded samples from further analyses. We measured RNA quantity and purity using a Nanodrop 8000 (ThermoScientific). When contamination was indicated by low 260/280 nm or 260/230 nm ratios, we purified the samples on RNeasy columns again. We then reverse transcribed 0.7 µg of RNA from each sample using Quantitect reverse transcription kits (Qiagen) and included controls without the reverse transcriptase. We checked these technical controls using qPCR on an ABI 7500 Fast real-time PCR system with at least two of the reference genes to ensure absence of genomic DNA. Reverse transcribed samples were only included in further analysis if control sample amplification signals were at least 10 cycles later than the positive controls. This corresponds to less than 0.1% of the signal in the reverse transcribed samples being due to contamination with genomic DNA and was considered acceptable.

After all control steps, 92 samples were left to measure expression levels of the target genes. We measured expression using one Fluidigm 96.96 Dynamic Array IFCs on the BioMark System using EvaGreen DNA Binding Dye (Biotium) according to the Advanced Development Protocol 14 (PN 100–1208 B) by Fluidigm. The expression values from the Fluidigm 96.96 chip were measured in triplicates and we used the average of each technical triplicate as the raw expression value (Ct). We selected immune genes of interest based on previous studies on immune gene expression in *B. terrestris* [15], [21], [22] and other organisms [23]–[26]; the selected genes were: PGRP-S3, PGRP-LC, BGRP1, BGRP2, hemomucin, pelle, relish, basket, hopscotch, abaecin, apidaecin, defensin, hymenoptaecin, TEPA, lysozyme3, transferrin, ferritin, jafrac, thioredoxin-dependent peroxide reductase, peroxiredoxin5, glutathione S-transferase, Dscam, argonaute, aubergine, serpin27a, catsup, punch, vitellogenin. In this selection, genes from the receptor, signaling and effector levels of the four classical insect immune pathways were included as well as general stress response genes, antiviral genes and genes involved in iron transport (typically relevant for bacterial infections), reactive oxygen species regulation and metabolism regulation. NCBI accession numbers, primer sequences and gene descriptions for all genes can be found in Table S2 in File S1.

Published primers were used for hemomucin and relish [22], vitellogenin [27] and for ITPR [21]. We designed all other primers based on the GenBank sequences (Table S2 in File S1) in Primer3 [28] or Quantprime [29] to be 20±2 bp long and have a melting temperature of 60° ±1°C with a maximum of 0.5°C difference in melting temperature between forward and reverse primers. We tested all primers in real time PCR reactions on several samples with an annealing temperature of 60°C. Only primers with good specificity, reliability and amplification efficiency between 1.9 and 2.1 at this setting were used in the final experiment. Further details on primer tests can be found in Table S3 in File S1 and information on reference gene use in Table S4 in File S1.

### Statistical Analyses

To describe the different patterns in gene expression produced upon parasite exposure, colony background and collection sites, we performed two kinds of statistical analyses: first, we assessed the effects of our experimental factors “parasite exposure”, “site” and “colony” on the full set of genes combined and on each gene individually by M/ANOVA analyses. These analyses identify the factors and interactions which play an important role for general immune gene expression patterns and individual genes.

Second, we performed linear discriminant analyses (LDA) for group separation, *i.e.* among groups according to each of the experimental factors which were identified as significant influences on gene expression patterns by the MANOVA analysis. An LDA identifies the linear combinations of variables (genes in our case) that provide the best discrimination of the groups. The number of linear discriminant (LD) functions identified in a given LDA is N−1 with N being the number of different treatment groups for the analyzed factor. The N functions are ordered in descending weight to explain the separation of groups. In graphical representations, typically values along the two first functions (“axes”) are plotted, which together explain more of the separation than any subsequent function. From the LD coefficients, especially those pertaining to the first few LD-functions, we can thus tell which group of genes best describes the group separation. As the LD analyses introduce different dimensions with axes along which groups are maximally separated, the LD coefficients are not *per se* informative for gene up or down regulation; rather the absolute value of the (standardized) expression (independent of sign) is taken and thus indicates the relative contribution of the respective gene to group separation. LDA is therefore a useful follow-up analysis when significant effects are found in a MANOVA to identify the variable combinations that explain the multivariate effect best, whereas the univariate results of the ANOVA describes treatment effects on individual response variables, independent of the other response variables [30]. Finally, to explore the variation itself, we tested for differences in standard deviation of the dCt expression values of bees exposed to the parasite versus control bees, and across collection sites using separate Wilcoxon signed rank tests.

We performed all statistical analyses in R 2.13.1 [31] using dCt values as recommended by Yuan *et al.* [32]. For M/ANOVA analyses and discriminant analyses, data was Yeo-Johnson transformed within genes to improve normality and homoscedasticity of the data groups. The lambda values can be found in Table S5 in File S1. We used classical MANOVA to analyze the overall effects of the combined gene set and to yield univariate results for individual genes (base package in R). “Site” and “exposure” status of each sample were used as fixed factors, “colony” as a nested factor within site, and the transformed dCt values of the samples as the response variables. The degrees of freedom varied to a small extent because of missing data (samples excluded after control steps or reactions failed on the Fluidigm chip). For the linear discriminant analyses, we set the Yeo-Johnson transformed dCt values for expression of all genes as predictor variables and either exposure status, site of origin or colony as predicted group value using the MASS package [33]. To assess the prediction quality of the obtained LD functions, we performed leave-one-out cross-validation and calculated the percentage of cases classified correctly.

**Figure 1:**
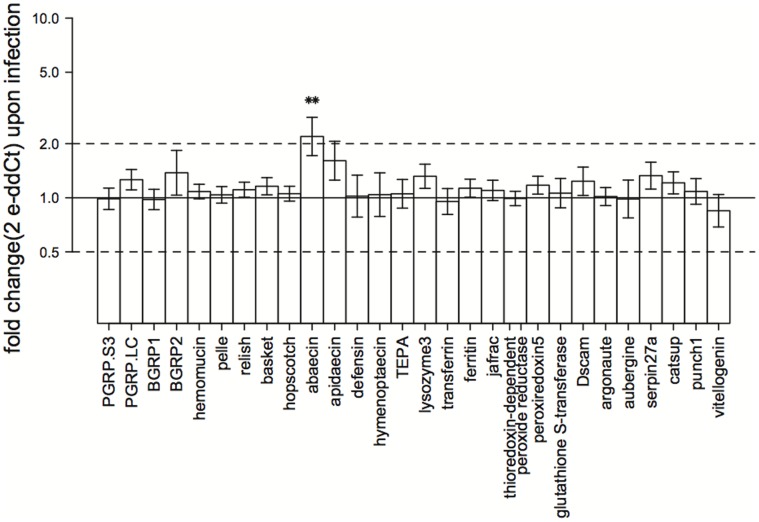
Gene expression changes upon infection. Presented values are calculated with the 2^-ddCt^ method. This method yields fold-change values for gene expression between defined sample groups (exposed compared to non-exposed). Error bars are standard errors calculated upon averaging dCt values within sample groups and transformed to fold change errors with error propagation. The solid line marks the value 1 and corresponds to no change between groups. Dashed lines mark the values 2 and 0.5, corresponding to doubled and halved gene expression upon treatment, respectively. Asterisks mark significance of effects as detectable in the univariate outputs of the overall MANOVA (Table S6a in File S1). Visualization of fold changes within the two collection sites can be found in Figure S1.

**Figure 2:**
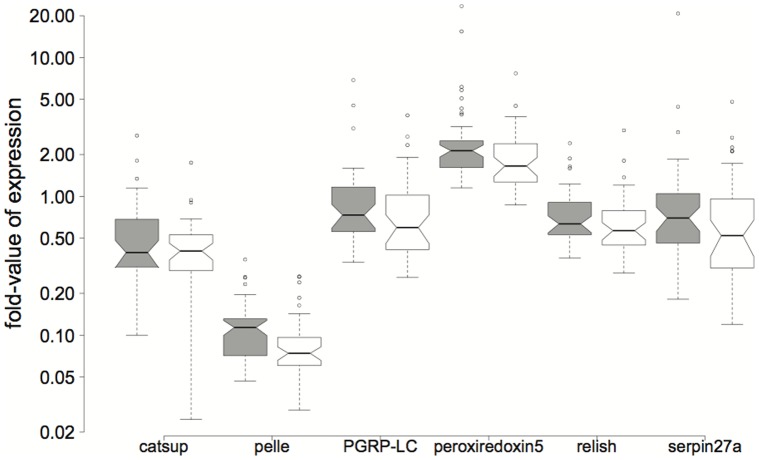
Boxplots of gene expression-fold values for catsup (*F*_1,77_ = 4.253, *P* = 0.043), pelle (*F*_1,77_ = 10.54, *P* = 0.002), PGRP-LC (*F*_1,77_ = 5.898, *P* = 0.017), peroxiredoxin5 (*F*_1,77_ = 11.64, *P* = 0.001), relish (*F*_1,77_ = 5.381, *P* = 0.023) and serpin27a (*F*_1,77_ = 4.075, *P* = 0.047). Neunforn results are presented in the left boxplot of each pair (in grey). All depicted genes are significantly different in expression between sites (Table S6a in File S1). Fold-expression values were calculated with dCt values (see main text) and are therefore on a scale defined by reference gene expression.

To improve graph tangibility, we used fold-expression values for result visualizations as recommended by Schmittgen & Livak [34]: fold expression = 2^−dCt^. It is important to note that the fold-expression presented in Fig. 1 is relative to control samples but Fig. 2 is relative only to reference gene levels.

## Results

### Parasite Exposure Effects

Exposure to *C. bombi* alters gene expression overall (Table 2, S6a). When we analyzed the effect of exposure in the different sites separately we found that exposure only significantly altered expression in colonies collected from Neunforn (Table 2, S6b). However, overall immune regulation patterns show similar tendencies, suggesting that differences between sites are caused by differences in the magnitude of gene expression rather than fundamentally different regulation of these genes in response to exposure to *C. bombi* (Fig. S1 in File S1).

**Table 2:** MANOVA results.

Multivariate effects full data set						
factor	Df	Pillai’s	F value	Num Df	Den Df	P-value
		trace				
**site**	1	0.667	3.382	29	49	*<* **0.001**
**infection**	1	0.548	2.052	29	49	**0.013**
**site:colony**	6	3.806	3.229	174	324	*<* **0.001**
site×infection	1	0.456	1.417	29	49	0.138
Residuals	77					
**Multivariate effects for site “Aesch”**						
**factor**	**Df**	**Pillai’s**	**F value**	**Num Df**	**Den Df**	**P-value**
		trace				
infection	1	0.894	2.323	29	8	0.107
**colony**	3	2.655	2.65	87	30	**0.002**
infection×colony	3	2.335	1.211	87	30	0.282
Residuals	36					
**Multivariate effects for site “Neunforn”**						
**factor**	**Df**	**Pillai’s**	**F value**	**Num Df**	**Den Df**	**P-value**
		trace				
**infection**	1	0.947	4.309	29	7	**0.026**
**colony**	3	2.803	4.413	87	27	*<* **0.001**
infection×colony	3	2.202	0.856	87	27	0.712
Residuals	35					

MANOVA was carried out on full data set of dCt values after Yeo-Johnson transformation for each gene and within data subsets according to collection site of queens. Transformation values can be found in Table S5 in File S1. The full MANOVA results including univariate effects can be found in Table S6 in File S1. As colonies are nested within sites, the site-colony interaction depicts the colony effect. Effects that are statistically significant (*P*<0.05) are highlighted in boldface.

Discriminant analysis shows that expression of the genes for the receptor PGRP-LC, the signaling molecules hopscotch, pelle, and relish, the antimicrobial peptide abaecin, and the enzymes jafrac and peroxiredoxin5 combined best describes the differences between the exposed and non-exposed groups (Table 3).

**Table 3:** Linear discriminant analyses for the factors site, infection and colony.

Grouping factor	Gene	LD coefficient	
site	peroxiredoxin5	4.969	
	hopscotch	−2.643	
	ferritin	−2.021	
	BGRP1	1.488	
infection	PGRP-LC	−3.761	
status	hopscotch	−2.4	
	abaecin	−1.732	
	jafrac	1.541	
	pelle	1.47	
	peroxiredoxin5	−1.439	
	relish	1.34	
**Grouping factor**	**Linear discriminant function**	**Proportion of trace**	**Genes with highest LD coefficients**
colony	LD1	0.391	basket, peroxiredoxin5, jafrac, hopscotch
	LD2	0.223	PGRP-S3, jafrac, basket, hopscotch
	LD3	0.149	peroxiredoxin5, hopscotch, PGRP-S3, basket
	LD4	0.092	PGRP-LC, peroxiredoxin5
	LD5	0.087	basket, PGRP-S3, jafrac
	LD6	0.04	hopscotch
	LD7	0.018	

R Code and the full set of LD coefficients can be found in Table S7 in File S1. Here we present only the genes with a coefficient greater than 1.1 for the site and infection effects and 2.0 for the colony effect. The magnitude of the linear discriminant coefficients indicates to what extent each factor (in this case: each gene) contributes to the predictive value of the linear discriminant function. The proportion of trace reports the predictive value of a linear discriminant function relative to the other LD functions when more than two groups are predicted and (N−1) LD functions are generated by the LDA (N being the number of groups). Leave-one-out cross validation accurately assigned samples to the correct site, infection condition, and colony 64.4%, 63.2%, and 55.2% respectively (as compared to the probabilities of 50%, 50%, and 12.5% as predicted by chance).

In univariate ANOVA analyses, a significant effect of *C. bombi* exposure was detectable only for abaecin (*F*_1,77_ = 11.592, *P* = 0.001) (Table S6a in File S1 and Fig. 1), and PGRP-S3 is expressed differently upon exposure depending on the collection site of the bees (*F_1,77_* = 4.043, *P* = 0.048, Table S6 in File S1). Parasite exposure significantly also increased the variation in gene expression (V = 290, *P = *0.024).

### Collection Site Effects

Bees from the two collection sites differ in their expression of several genes. The enzyme peroxiredoxin5, the signaling molecule hopscotch, the iron transportation protein ferritin, and the receptor BGRP1 differed among locations according to LDA (Tables 3, S7). When analyzing the expression of genes individually, the expression of catsup (*F*_1,77_ = 4.253, *P* = 0.043), pelle (*F*_1,77_ = 10.54, *P* = 0.002), PGRP-LC (*F*_1,77_ = 5.898, *P* = 0.017), peroxiredoxin5 (*F*_1,77_ = 11.64, *P* = 0.001), relish (*F*_1,77_ = 5.381, *P* = 0.023) and serpin27a (*F*_1,77_ = 4.075, *P* = 0.047) varied significantly across collection locations (Table S6a in File S1). All of these genes are more strongly expressed in bees from Neunforn colonies than in bees from Aesch colonies (Fig. 2) but expression variance is not significantly different between populations (V = 234, *P* = 0.247). Neither the parasite exposure effect nor the site differences give patterns distinct enough to assign individuals reliably into their groups with the discriminant functions (64.4% of individuals correctly predicted for site origin and 63.2% for infection status with a 50% probability of correct assignment by chance in each case).

### Colony Effects

**Figure 3:**
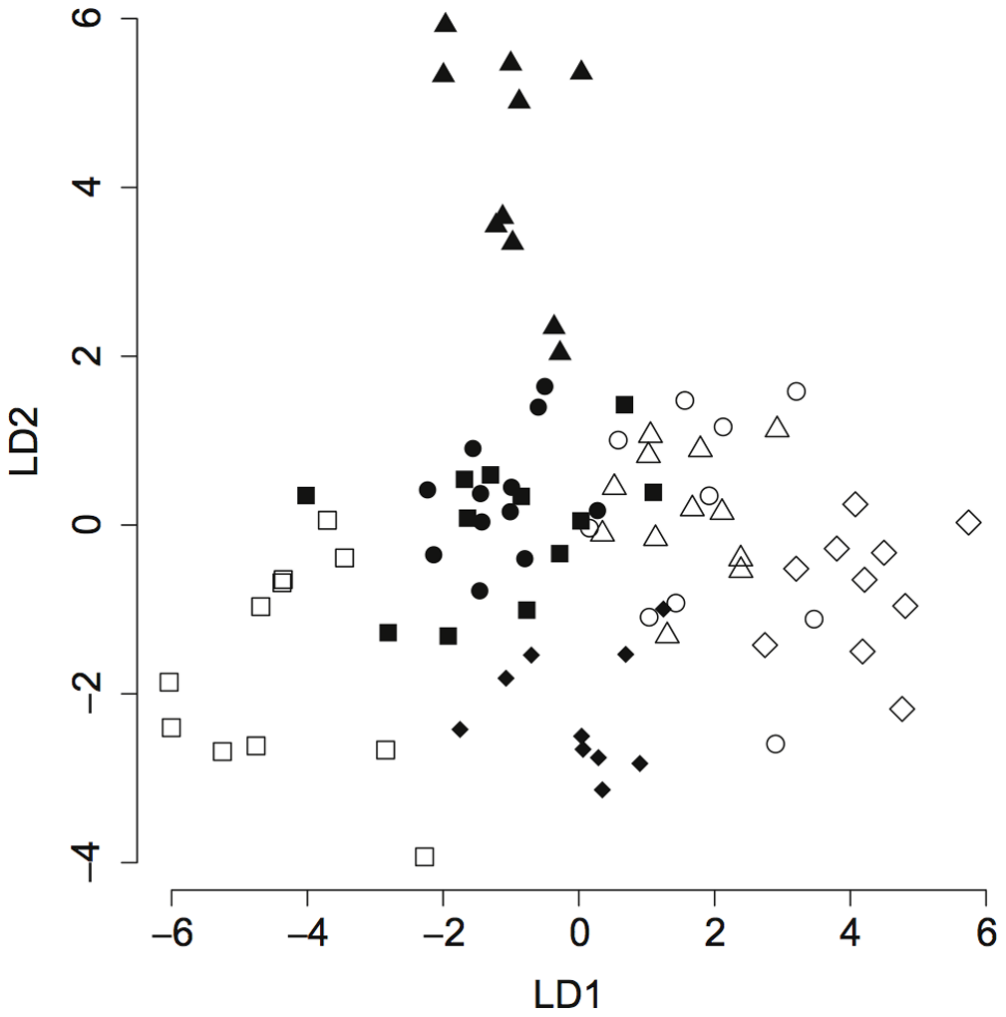
Discriminant analysis of gene expression by colony origin. Shown are individuals from colonies from site Aesch (A1 to A4, represented by filled diamonds, triangles, circles and squares, respectively) and Neunforn (N1 to N4, represented by open diamonds, triangles, circles and squares). LD1 and LD2 represent the first two linear discriminant functions. The main contributions to LD1 come from the genes basket, peroxiredoxin5, jafrac and hopscotch. Within LD2, PGRP-S3, jafrac, basket and hopscotch have the highest coefficients. Despite considerable variation within colonies, clusters are already visible and become fairly distinguishable when taking into account all 7 discriminant functions (55.2% of cases classified correctly as opposed to 12.5% expected by chance, Table 3).

The expression of immune genes varied strongly across colonies (Table 2). Almost all genes show a significant colony effect (univariate results in S6a) and the linear discriminant functions predict colony identity correctly in 55.2% of the cases. This is considerably higher than the 12.5% probability of assignment to the correct colony by chance (as there are 8 different colonies), suggesting that there are distinct immune profiles for each colony that can be described by the linear discriminators. Again, the main contributions to these profiles (as assessed by the linear discriminant coefficients) come from the signaling molecules hopscotch and basket, the receptor molecules PGRP-S3 and PGRP-LC, and from the enzymes peroxiredoxin5 and jafrac (Table 3). Despite high intra-colony variation (among workers) colony-specific profiles are distinguishable (Fig. 3).

## Discussion

Starting with our main question, we find that exposure to the parasite *Crithidia bombi* significantly influences *Bombus terrestris* immune gene expression. The effects of collection site and colony were more consistent and therefore showed up as stronger effects in the analyses of variance, but overall differences in immune gene expression upon parasite exposure remained visible despite significant variation among colonies, among individuals within colonies and between the two populations surveyed. This finding is ecologically (and evolutionarily) relevant, since few studies capture the breadth of variation in immune responses across a diverse range of immunologically important genes or account for variation on this scale. The variability in immunological responses to parasitism seen here and in previous studies of the same system [15], [22] emphasizes the importance for assessing gene expression patterns in a variety of genetic backgrounds if general effects are of interest rather than effects specific for one colony (or one host type or location). In fact, gene expression responses to *C. bombi* infections have been tested before but usually in small numbers of commercially reared colonies and using relatively few genes (Table 1). To our knowledge, there is also only one other study to date that used colonies from wild caught bees [22].

The different immune expression profiles among colonies (discriminant analysis in Table 3 and visualized in Fig. 3), irrespective of exposure status, are driven by genes that encode signaling molecules from the JNK and the JAK/STAT pathways (in the JNK: kinase basket; in the JAK: kinase hopscotch), receptor molecules from the NF-κB pathways (PGRP-S3 and PGRP-LC) and peroxiredoxins (peroxiredoxin5 and jafrac) that have antioxidant functions as well as possible immune-regulatory roles in *Drosophila* [24]. Given the proposed functions of these genes [35], this indicates that the main difference between colonies (for exposed and non-exposed bees combined) can be found at the level of immune system regulation, in the crosstalk of immune pathways, and in the regulation of reactive oxygen species (ROS) for gut homeostasis, rather than on the effector level (e.g. the anti-microbial peptides). When looked at independently, almost all of the genes in our set display a colony effect (Table S6 in File S1) but with our experimental design, we can not say whether this is due to genetic, epigenetic or environmental effects that have all been shown to influence infection dynamics of *C. bombi* in *B. terrestris* [12], [36]–[38] and that likely all contribute to differences in immune gene expression as well. The strong differences among colonies are important given the well-described genotype-by-genotype pattern of infection in this system. Some colonies of *B. terrestris* are infected by some clones of *C. bombi* but not others [11], [12]. The high variation we find here among colonies suggests that gene expression differences of immune genes may be important in producing this signal of host-parasite specificity.

The genes determining differences across sites (discriminant analysis in Table 3) are mostly related to homeostasis regulation (peroxiredoxin5 and the iron transport protein ferritin) and upstream actors of general immune pathways (the JAK pathway signaling molecule hopscotch and the NF-κB pathway receptor BGRP1). Interestingly, when testing expression of genes individually (Table S6a in File S1), other immune functions come into play: serpin27a (a serine protease inhibitor involved in prophenoloxidase (PPO) regulation [39]) and catsup (an enzyme involved in melanin synthesis in *Drosophila* [40]) are both differentially expressed across the two populations. The PPO-cascade ultimately leads to the activation of the melanization reaction [41], therefore both serpin27a and catsup indicate that the melanization immune reaction is important for the immunological difference between our two sites. As with the colony differences above, variation among sites could be driven by genetic, epigenetic or environmental differences. The sites Neunforn and Aesch differ in *Crithidia* infection prevalence (5% *vs.* 12.5% respectively between 2007–2011, Table S1 in File S1), which might translate into different selection regimes and/or cause different immune memory backgrounds in queens from the two sites and could potentially explain the differences we see in immune gene expression across sites.

Exposure to *C. bombi* altered gene expression even after conservative statistical analysis (i.e. partitioning variance first into the site in our hierarchical model leaving less residual variation to be partitioned into the exposure effect). In particular, we found that the antimicrobial effector abaecin was generally up regulated upon exposure (Table S6a in File S1), whereas the overall significant exposure effect was explained best by a combination of factors from different levels in the NF-κB and JAK/STAT pathways together with abaecin and the two peroxiredoxins jafrac and peroxiredoxin5 (LDA, Table 3). Our results suggest that all classic insect immune pathways (Toll, Imd, JNK and JAK/STAT) are involved in the response of *B. terrestris* to exposure to *C. bombi*. Furthermore, it is likely that the genes involved in ROS regulation are important both in the direct response to infection and in the general fine tuning of the immune system and gut homeostasis in *B. terrestris* - which is reflected in differences between colonies and sites. Peroxiredoxin5 appears to play an important role in the *B. terrestris* immune response to *C. bombi*. This gene was identified as a driver of all tested effects (site, exposure and colony, Table 3), shows generally very high expression levels (Fig. 2), and its expression differs significantly between sites (Table S6a in File S1). The general importance of peroxiredoxin5 is supported by the finding of Ha *et al.* [42] who found that the regulation of ROS can be more important for gut immunity than immune pathways producing antimicrobial peptides.

We did not detect up regulation of the pathogen receptor hemomucin and the signaling molecule relish as found by Schlüns *et al.* [22]. Most likely, this is due to differences in collection sites and years between these studies, as well as gene expression level assessment at different time points post infection. Additionally, our bees were not age-controlled as in the study of Schlüns *et al.* [22], in order to capture colony level variation, which adds further natural variation to gene expression and could mask effects that might be visible in single age classes. Riddell *et al.* [16] described up regulation of the antimicrobial peptides abaecin and defensin within 12 hours of *Crithidia* infection. We also found that abaecin was upregulated 18 hours post infection but did not detect up regulation of defensin. This is likely due to the assessment of 8 different colonies at the same time in our study, giving more general results, while Riddell *et al.* based their results on a single colony. Interestingly, Riddell *et al.* also describe high variation in immune gene expression among individuals, even within a colony, suggesting that this may be a common trait of *B. terrestris*.

These differences between the overall results of similarly designed studies emphasize again that immune gene expression has to be measured in a variety of samples with different genetic and ecological backgrounds before general conclusions can be drawn. Controlling for sources of natural variation is beneficial when looking for very specific effects and when trying to isolate and identify specific causal relationships. But conclusions from such experiments have to be drawn within this same, narrow framework. To find general effects, natural variation on all levels has to be taken into account. Such variation can also be ecologically important. This is certainly the case in the context of ecological immunology, considering the need for an evolutionary potential in the arms race of host and parasite. Altogether, studies covering a higher diversity of samples are more likely to be informative about processes under natural conditions. The fact that a variety of effects were still visible in our sample set despite the strong variation among individuals suggests that this method of gene expression measurement could be useful in diverse ecological contexts and even in field samples.

In summary, we have shown that the four classical insect immune pathways leading to the immune responses of melanization and antimicrobial peptide production are likely involved in the response of *B. terrestris* to the trypanosome *C. bombi* and that expression of genes governing immune responses vary greatly between and even within colonies. The influence of the sample collection site on both general expression levels and infection responses adds yet another level to these variation patterns. Gene expression differed among our collection sites and we suggest that this should be generally taken into consideration when designing gene expression experiments using samples from wild populations.

Our study also provides a good example of how microfluidic devices can facilitate the targeted investigation of gene expression patterns of non-model organisms like *B. terrestris* and provide enough power to identify patterns in gene expression through ecologically relevant levels of biological noise. Interesting questions emerging from our findings include the source and the potential benefits of the strong variation across individuals. We, and others [16], have found high levels of individual variation in both constitutive and induced forager immune gene expression. Whether this variation is in itself adaptive remains to be tested, for example in the context of an immunological division of labor. An immunologically heterogeneous environment will likely pose considerable challenges to parasites that invade, effectively limiting their available host population to some subset of the colony. While immunological diversity might increase the number of strains that are able to infect a colony and establish, it could also limit the total number of circulating strains to the number of immunological castes and prevent further strains from accumulating within the colony. Our finding of important site differences also leads to the question of possible local adaptation patterns in gene expression. The data from our diverse array of genes provides useful indications as to which genes might be interesting targets for future studies to answer these questions.

## Supporting Information

File S1 Contains data about the *C. bombi* infection prevalence in the wild, additional statistical results, primer details, Tables S1–S7 and Fig. S1.
(DOCX)
