# Supplementary material for: Immune Gene Expression in Bombus terrestris: Signatures of Infection Despite Strong Variation among Populations, Colonies, and Sister Workers
Source: PLoS One. 2013 Jul 15;8(7):e68181. doi: 10.1371/journal.pone.0068181 (PMC3712019; doi:10.1371/journal.pone.0068181)
Supplement: File S1 — Contains data about the C. bombi infection prevalence in the wild, additional statistical results, primer details, Tables S1–S7 and Fig. S1. (DOCX) [file pone.0068181.s001.docx]

Table S1 *Crithidia bombi* infection prevalence in *Bombus terrestris* spring queens

| Year | Percent of collected spring queens naturally infected with *Crithidia bombi* (N) | |
| --- | --- | --- |
|  | Aesch | Neunforn |
| 2011 | 4.673 (214) | 0 (190) |
| 2010 | 24.80 (125) | 6.616 (393) |
| 2009 | 13.71 (175) | 4.348 (161) |
| 2008 | 11.26 (151) | 11.64 (189) |
| 2007 | 8.176 (159) | 2.649 (151) |
| **Average** | **12.52** | **5.051** |

| *Crithidia* prevalence in Aesch is significantly higher in a one sided paired t-test (t_4_ = 2.415, *P*= 0.037) |
| --- |

| Table S2 Surveyed genes and primer specifications | | | | | | |
| --- | --- | --- | --- | --- | --- | --- |
| **Gene** | **Putative gene function** | **NCBI accession** | **Forward primer** | **Reverse primer** | **Product size** | **Primer Reference** |
| **PGRP-S3** | recognition receptor, Toll pathway | XM_003401893 | CGTGAAGGAGCTCATACCAT | CCAGGACTCATAGTGGCTGT | 200 |  |
| **PGRP-LC** | recognition receptor, Imd pathway | XM_003396463 | CAGCCACCTACGACAGATTT | GTACATTCCGCTTGTGTCCT | 101 |  |
| **BGRP1** | recognition receptor, Toll pathway | XM_003397996 | AACGTGGAAGTCAAAGATGG | GCGAACGATGACTTGGTATT | 206 |  |
| **BGRP2** | recognition receptor, Toll pathway | XM_003394713 | TAACTCCCTTTGGAAACACG | GGCGGTAAAATACTGAACGA | 249 |  |
| **hemomucin** | surface glycoprotein, potential recognition receptor | XR_131963 | AGCATTCCCAGATTTAGCACT | TAACAGTTGATTTCGGAGGTA | 173 | Schlüns *et al* ([2010](#_ENREF_4)) |
| **pelle** | signal molecule, Toll pathway | XM_003399470 | TAAATCGACCTATGCAAGCC | GGGTATAGCTGCTTCTGCTG | 107 |  |
| **relish** | signal molecule, Imd pathway | XM_003399472 | CAGCAGTAAAAATCCCCGAC | CAGCACGAATAAGTGAACATA | 156 | Schlüns *et al* ([2010](#_ENREF_4)) |
| **basket** | signal molecule, JNK pathway | XM_003402794 | GGAACAAGATAATCGAGCAACTG | CTGGCTTTCAATCGGTTGTG | 177 |  |
| **hopscotch** | signal molecule, JAK/STAT pathway | XM_003401903 | CACAGACTGAAGCAGGTTGA | CATATGGGTAATTTGGTGCC | 353 |  |
| **abaecin** | antimicrobial peptide (AMP) | XM_003394653 | GCCACAATATGTGGAATCCT | ATGACCAGGGTTTGGTAATG | 141 |  |
| **apidaecin** | antimicrobial peptide (AMP) | XM_003402966 | CCCGACTAATGTACCTGCCA | GAAGGTGCGAATGTGTTGGA | 131 |  |
| **defensin** | antimicrobial peptide (AMP) | XM_003395924 | GTCTGCCTTTGTCGCAAGAC | GACATTAGTCGCGTCTTCTTCG | 139 |  |
| **hymenoptaecin** | antimicrobial peptide (AMP) | XR_132450 | TTCATCGTACTGGCTCTCTTCTG | AGCCGTAGTATTCTTCCACAGC | 85 |  |
| **TEPA** | effector molecule, JAK/STAT pathway | XM_003399699 | GCGTTCTATGACCACCTGTT | TACAGGTTACTCCACAGCCC | 212 |  |
| **lysozyme3** | bacteriolytic effector | XM_003394052 | TATGGGCAAGAAGATTCGAC | GTGTACATCGTTCACGCATC | 219 |  |
| **transferrin** | iron-binding protein, antibacterial | XM_003401163 | CAATTTCTTCACCGCATCCT | CCTCGTTATTTGGCTTGCAT | 131 |  |
| **ferritin** | iron transportation protein | XM_003393332 | AAAGAATTGGACGCAAATGG | CAGCGAACTGATGTCCAAGA | 259 |  |
| **jafrac** | peroxiredoxin, ROS regulation | XM_003401245 | CTCACTTCAGTCACTTGGCA | GCCAGCAGGACATACTTCTC | 290 |  |
| **thioredoxin-dependent peroxide reductase** | ROS regulation | XM_003401316 | GCCGTGGTTGATGGTGATTTC | CAGTGGGACACACAAATGTGAAG | 106 |  |
| **peroxiredoxin5** | peroxiredoxin, ROS regulation | XM_003394777 | TCACACCAGGATGTTCCAAGAC | TTCTGCTCCGTGTTCTTTACCC | 146 |  |
| **glutathione S-transferase** | ROS regulation | XM_003403373 | CAGTCGCTGAGAGAACATCAAG | GTAACTTCCGGCCACTCTTTC | 78 |  |
| **dscam** | phagocytosis of pathogens | XM_003394934 | GAGCGAGGAACGAAGACAAC | CCGTTTGGCAGTGAATAGGT | 294 |  |
| **argonaute** | RNA-interference, possible antiviral function | XM_003398481 | AATTGCAAGATCAACCTGCC | CCTACCCAAAGACAAGGCAA | 175 |  |
| **aubergine** | RNA-interference, possible antiviral function | XM_003400641/XM_003400642 | GTCGCCCTTCTGCATATCTC | AAGATCGAACTGCTATCCGC | 190 |  |
| **serpin27a** | serine protease inhibitor, PPO cascade | XM_003392985 | CCGATCATCCATTCGTATTC | ACCTGCACTTGATATCCCTG | 164 |  |
| **catsup** | enzyme, melanin synthesis | XM_003398173 | TTACCATGACGAGTCACCAA | ATGAGGAACCAAAGCATGAG | 355 |  |
| **punch** | enzyme, melanin synthesis | XR_131852 | ATTGCCAGGACACTTTCAAC | TACAAGCTGGAAACGGAAAC | 211 |  |
| **vitellogenin** | various metabolic and endocrinological functions | XM_003402655/XM_003402656 | GTGACAAGCGAAGAGACTATTATG | CCGTGTTATCTGGCGTGAC | 154 | Li *et al*  ([2010](#_ENREF_2)) |
| **AK** | arginine kinase, housekeeping | AF_492888 | CTGGACTCTGGTGTCGGTAT | GTCTTTTGGTGGATGCTTGT | 129 |  |
| **PLA2** | phospholipase A2, housekeeping | FN_391388 | TATCTTTCAATGCCCAGGAG | GTCGTAACAAATGTCATGCG | 129 |  |
| **ITPR** | inositol 1,4,5-trisphosphate, housekeeping | DQ_468668 | TGCACGCAGACCAAGCGGAG | ACGTCTTCCTTCGCGTCAAACGG | 190 | Erler *et al (*[*2011*](#_ENREF_1)*)* |

Table S3 Primer tests carried out on 96 well plates in 7500 FAST Real Time PCR System (ABI) with EvaGreen chemistry (biotium)

| **Testing primer reliability** | All primers were tested on several samples, with several cDNA dilution ratios and including technical replicates. Only primers with reliable amplification curves were used further in the experiments. Furthermore, negative controls without template were run with the pretests to compare primerdimer melt curves and target melt curves. These had to be clearly different to ensure that the sample well signals were not confounded with primerdimer signals.  The threshold for primer acceptance was set to a difference of 6 cycles between the highest Ct values from samples and the Ct values of negative controls. This ensures that a maximum of 1.6% of the signal in the sample wells could be due to primerdimer formation. Also, primers with primerdimer signal before cycle 30 in the 7500 ABI qPCR reaction were excluded from the primer set. |
| --- | --- |
| **Testing primer specificity** | The primer products (amplified from a pool of cDNA from different samples) were sequenced on a 3130xl DNA Analyzer (ABI) and BLASTed. Correct targeting of the gene of interest could be confirmed for all primers except apidaecin which consistently failed to sequence.  Meltcurves of primers were checked for several samples in the pretests and for all Fluidigm reactions as well. Primers with ambiguous melt curves were excluded from the dataset. |
| **Testing primer efficiency** | Primer efficiencies were assessed for all test reactions using the program LinReg ([Ramakers *et al.* 2003](#_ENREF_3))  Primers with efficiencies above 2.1 or below 1.9 were excluded from further experiments, as well as primers with strong variation of efficiencies between technical replicates, different cDNA dilution ratios or between different samples. Efficiencies of primers on the Fluidigm chip could not be determined as melt curve resolution was not sufficient. |

Table S4 Reference gene use

| AK, PLA2 and ITPR were measured as references genes as recommended by Hornakova *et al.* (2010) and as used in previous gene expression studies on *B. terrestris* |
| --- |
| GeNorm analysis on the chip data in qbasePLUS (biogazelle) suggested AK and PLA2 as the most stable reference gene combination and the geometric mean of their measured expression values did not vary significantly with the experimental factors (ANOVA: site *F*_1,88_ = 0.4847, *P* = 0.4881; infection status *F*_1,88_ = 0.3283, *P*=0.5681; site x infection status *F*_1,88_ = 1.1577, *P* = 0.2849) |
| This combination of AK and PLA2 was therefore used as the reference value (RV) to normalize the raw expression data (Ct) between samples as recommended by Vandesompele *et al* (2002):  RV=  |

Table S5 *λ* values used for Yeo-Johnson transformation of dCt datasets

| **gene** | **λ** |
| --- | --- |
| PGRP-S3 | 1.86994 |
| PGRP-LC | 1.588561 |
| BGRP1 | 1.34755 |
| BGRP2 | 0.203494 |
| hemomucin | no transformation |
| pelle | no transformation |
| relish | 1.760138 |
| basket | 0.5134408 |
| hopscotch | no transformation |
| abaecin | 1.432074 |
| apidaecin | 1.316675 |
| defensin | 1.225636 |
| hymenoptaecin | 1.6847 |
| TEPA | no transformation |
| lysozyme 3 | 1.808369 |
| transferrin | no transformation |
| ferritin | no transformation |
| jafrac | 1.249531 |
| thioredoxin-dependent peroxide reductase | 2.295916 |
| peroxiredoxin5 | 2.258144 |
| glutathione S-transferase | 1.668058 |
| Dscam | 1.689414 |
| argonaute | 1.556782 |
| aubergine | 1.205779 |
| serpin27a | 1.401884 |
| catsup | no transformation |
| punch | 1.347483 |
| vitellogenin | no transformation |

| Table S6a MANOVA results: MANOVA was carried out on full data set of dCt values after Yeo-Johnson transformation for each gene. | | | | | | | |
| --- | --- | --- | --- | --- | --- | --- | --- |
| **Used R Code** | | | | | | | |
| fit1 <- manova(transformed.dCt.values ~ site+colony%in%site+infection+infection:site)  manova.output <- summary(fit1,test=c("Pillai"))  anova.output <- summary.aov(fit1) | | | | | | | |
|  | | | | | | | |
| **Multivariate effects** | | | | | | | |
| factor | Df | Pillai’s trace | F value | | num Df | den Df | P-value |
| **site** | 1 | 0.667 | 3.382 | | 29 | 49 | **< 0.001** |
| **infection** | 1 | 0.548 | 2.052 | | 29 | 49 | **0.013** |
| **site:colony** | 6 | 3.806 | 3.229 | | 174 | 324 | **< 0.001** |
| site x infection | 1 | 0.456 | 1.417 | | 29 | 49 | 0.138 |
| Residuals | 77 |  |  | |  |  |  |
|  |  |  |  | |  |  |  |
| **Univariate effects** | | | | | | | |
| gene | | factor | | F value | | P-value | |
| **PGRP-S3** | | site | | *<*0.001 | | 0.982 | |
|  | | infection | | 0.560 | | 0.457 | |
|  | | site:colony | | 2.731 | | **0.019** | |
|  | | site x infection | | 4.043 | | **0.048** | |
| **PGRP-LC** | | site | | 5.898 | | **0.017** | |
|  | | infection | | 1.943 | | 0.167 | |
|  | | site:colony | | 3.144 | | **0.008** | |
|  | | site x infection | | 0.368 | | 0.546 | |
| **BGRP1** | | site | | 0.976 | | 0.326 | |
|  | | infection | | 0.003 | | 0.954 | |
|  | | site:colony | | 5.078 | | ***<*0.001** | |
|  | | site x infection | | 3.357 | | 0.071 | |
| **BGRP2** | | site | | *<*0.001 | | 0.991 | |
|  | | infection | | 2.475 | | 0.120 | |
|  | | site:colony | | 10.29 | | ***<*0.001** | |
|  | | site x infection | | 1.032 | | 0.313 | |
| **hemomucin** | | site | | 3.176 | | 0.079 | |
|  | | infection | | 0.582 | | 0.448 | |
|  | | site:colony | | 3.971 | | **0.002** | |
|  | | site x infection | | 0.498 | | 0.482 | |
| **pelle** | | site | | 10.54 | | **0.002** | |
|  | | infection | | 0.088 | | 0.767 | |
|  | | site:colony | | 2.844 | | **0.015** | |
|  | | site x infection | | 0.081 | | 0.777 | |
| **relish** | | site | | 5.381 | | **0.023** | |
|  | | infection | | 0.112 | | 0.739 | |
|  | | site:colony | | 3.365 | | **0.005** | |
|  | | site x infection | | 1.699 | | 0.196 | |
| **basket** | | site | | 0.096 | | 0.757 | |
|  | | infection | | 0.662 | | 0.418 | |
|  | | site:colony | | 1.964 | | 0.081 | |
|  | | site x infection | | 1.301 | | 0.258 | |
| **hopscotch** | | site | | 0.261 | | 0.611 | |
|  | | infection | | 0.622 | | 0.433 | |
|  | | site:colony | | 6.238 | | *<***0.001** | |
|  | | site x infection | | 3.045 | | 0.085 | |
| **abaecin** | | site | | 0.015 | | 0.904 | |
|  | | **infection** | | 11.59 | | **0.001** | |
|  | | **site:colony** | | 3.690 | | **0.003** | |
|  | | site x infection | | 1.692 | | 0.197 | |
| **apidaecin** | | site | | 0.041 | | 0.840 | |
|  | | infection | | 3.831 | | 0.054 | |
|  | | **site:colony** | | 4.907 | | ***<*0.001** | |
|  | | site x infection | | 2.654 | | 0.107 | |
| **defensin** | | site | | 0.068 | | 0.794 | |
|  | | infection | | *<*0.001 | | 0.993 | |
|  | | site:colony | | 1.909 | | 0.090 | |
|  | | site x infection | | 0.728 | | 0.396 | |
| **hymenoptaecin** | | site | | 1.777 | | 0.187 | |
|  | | infection | | 0.117 | | 0.734 | |
|  | | site:colony | | 1.667 | | 0.141 | |
|  | | site x infection | | <0.001 | | 0.979 | |
| **TEPA** | | site | | 2.927 | | 0.091 | |
|  | | infection | | 0.796 | | 0.375 | |
|  | | site:colony | | 2.400 | | **0.035** | |
|  | | site x infection | | 1.026 | | 0.314 | |
| **lysozyme 3** | | site | | 0.003 | | 0.954 | |
|  | | infection | | 2.167 | | 0.145 | |
|  | | site:colony | | 3.191 | | **0.008** | |
|  | | site x infection | | 2.809 | | 0.098 | |
| **transferrin** | | site | | 1.349 | | 0.249 | |
|  | | infection | | 0.015 | | 0.902 | |
|  | | site:colony | | 6.846 | | *<***0.001** | |
|  | | site x infection | | 2.636 | | 0.109 | |
| **ferritin** | | site | | 0.539 | | 0.465 | |
|  | | infection | | 0.007 | | 0.936 | |
|  | | site:colony | | 4.648 | | *<***0.001** | |
|  | | site x infection | | 0.022 | | 0.883 | |
| **jafrac** | | site | | 1.990 | | 0.162 | |
|  | | infection | | 0.064 | | 0.801 | |
|  | | site:colony | | 3.809 | | **0.002** | |
|  | | site x infection | | 1.413 | | 0.238 | |
| **thioredoxin dependent** | | site | | 3.844 | | 0.054 | |
| **peroxide reductase** | | infection | | 1.047 | | 0.309 | |
|  | | site:colony | | 2.873 | | **0.014** | |
|  | | site x infection | | 1.480 | | 0.228 | |
| **peroxiredoxin5** | | site | | 11.64 | | **0.001** | |
|  | | infection | | 0.864 | | 0.356 | |
|  | | site:colony | | 4.010 | | **0.002** | |
|  | | site x infection | | 1.466 | | 0.230 | |
| **glutathione S- transferase** | | site | | 1.906 | | 0.171 | |
|  | | infection | | 0.019 | | 0.890 | |
|  | | site:colony | | 2.214 | | 0.050 | |
|  | | site x infection | | 0.854 | | 0.358 | |
| **Dscam** | | site | | 2.643 | | 0.108 | |
|  | | infection | | 0.960 | | 0.330 | |
|  | | site:colony | | 3.268 | | **0.006** | |
|  | | site x infection | | 0.142 | | 0.707 | |
| **argonaute** | | site | | 3.612 | | 0.061 | |
|  | | infection | | 0.231 | | 0.6324274 | |
|  | | site:colony | | 4.601 | | ***<*0.001** | |
|  | | site x infection | | 0.099 | | 0.754 | |
| **aubergine** | | site | | 1.025 | | 0.314 | |
|  | | infection | | 0.013 | | 0.909 | |
|  | | site:colony | | 2.838 | | **0.015** | |
|  | | site x infection | | 0.579 | | 0.4489 | |
| **serpin27a** | | site | | 4.076 | | **0.047** | |
|  | | infection | | 1.445 | | 0.233 | |
|  | | site:colony | | 4.882 | | *<***0.001** | |
|  | | site x infection | | 2.375 | | 0.127 | |
| **catsup** | | site | | 4.253 | | **0.043** | |
|  | | infection | | 3.026 | | 0.086 | |
|  | | site:colony | | 4.811 | | ***<*0.001** | |
|  | | site x infection | | 0.662 | | 0.419 | |
| **punch** | | site | | 3.954 | | 0.050 | |
|  | | infection | | 0.002 | | 0.966 | |
|  | | site:colony | | 5.528 | | *<***0.001** | |
|  | | site x infection | | 0.399 | | 0.530 | |
| **vitellogenin** | | site | | 1.875 | | 0.175 | |
|  | | infection | | 0.601 | | 0.441 | |
|  | | site:colony | | 5.383 | | *<***0.001** | |
|  | | site x infection | | 0.001 | | 0.972 | |

Table S6b MANOVA results: two MANOVAs were carried out on site subsets of dCt values after Yeo-Johnson transformation for each gene

| **Used R Code** | | | | | | | | | | |
| --- | --- | --- | --- | --- | --- | --- | --- | --- | --- | --- |
| fit2 <- manova(transformed.dCt.values.Aesch ~ infection*colony)  manova.output <- summary(fit2,test=c("Pillai"))  anova.output <- summary.aov(fit2)  fit3 <- manova(transformed.dCt.values.Neunforn ~ infection*colony)  manova.output <- summary(fit3,test=c("Pillai"))  anova.output <- summary.aov(fit3) | | | | | | | | | | |
|  | | | | | | | | | | |
| **Multivariate effects Aesch** | | | | | | | | | | |
| factor | | Df | Pillai’s trace | F-value | | | num Df | den Df | | P-value |
| infection | | 1 | 0.894 | 2.323 | | | 29 | 8 | | 0.107 |
| **colony** | | 3 | 2.655 | 2.650 | | | 87 | 30 | | **0.002** |
| infection x colony | | 3 | 2.335 | 1.211 | | | 87 | 30 | | 0.282 |
| Residuals | | 36 |  |  | | |  |  | |  |
| **Multivariate effects Neunforn** | | | | | | | | | | |
| factor | | Df | Pillai’s trace | F-value | | | num Df | den Df | | P-value |
| **infection** | | 1 | 0.947 | 4.309 | | | 29 | 7 | | **0.026** |
| **colony** | | 3 | 2.803 | 4.413 | | | 87 | 27 | | ***<*0.001** |
| infection x colony | | 3 | 2.202 | 0.856 | | | 87 | 27 | | 0.712 |
| Residuals | | 35 |  |  | | |  |  | |  |
| Effects significant at *α* = 5% are highlighted in bold | | | | | | | | | | |
|  | | | | | | | | | | |
| **Univariate effects** | | | | | | | | | | |
| gene | factor | | | | F-value  Aesch | P-value  Aesch | | | F-value  Neunforn | P-value  Neunforn |
| **PGRP-S3** | infection | | | | 4.609 | **0.039** | | | 0.524 | 0.474 |
|  | colony | | | | 4.348 | **0.010** | | | 1.651 | 0.195 |
|  | infection x colony | | | | 0.753 | 0.528 | | | 0.045 | 0.987 |
| **PGRP-LC** | infection | | | | 0.146 | 0.704 | | | 3.193 | 0.083 |
|  | colony | | | | 2.509 | 0.074 | | | 3.965 | **0.016** |
|  | infection x colony | | | | 0.887 | 0.457 | | | 0.426 | 0.735 |
| **BGRP1** | infection | | | | 2.036 | 0.162 | | | 2.411 | 0.129 |
|  | colony | | | | 4.529 | **0.009** | | | 5.113 | **0.005** |
|  | infection x colony | | | | 0.732 | 0.540 | | | 0.916 | 0.443 |
| **BGRP2** | infection | | | | 0.019 | 0.891 | | | 5.243 | **0.028** |
|  | colony | | | | 9.184 | **<0.001** | | | 10.81 | *<***0.001** |
|  | infection x colony | | | | 0.190 | 0.903 | | | 1.156 | 0.340 |
| **hemomucin** | infection | | | | <0.001 | 0.993 | | | 1.320 | 0.258 |
|  | colony | | | | 3.008 | **0.043** | | | 4.837 | **0.006** |
|  | infection x colony | | | | 0.554 | 0.649 | | | 0.495 | 0.688 |
| **pelle** | infection | | | | 0.211 | 0.648 | | | 0.005 | 0.942 |
|  | colony | | | | 1.032 | 0.390 | | | 6.131 | **0.002** |
|  | infection x colony | | | | 3.225 | 0.034 | | | 0.586 | 0.628 |
| **relish** | infection | | | | 0.604 | 0.442 | | | 2.214 | 0.146 |
|  | colony | | | | 3.314 | **0.031** | | | 3.645 | **0.022** |
|  | infection x colony | | | | 2.606 | 0.067 | | | 0.341 | 0.795 |
| **basket** | infection | | | | 0.177 | 0.677 | | | 2.392 | 0.131 |
|  | colony | | | | 4.279 | **0.011** | | | 0.440 | 0.726 |
|  | infection x colony | | | | 3.633 | **0.022** | | | 2.086 | 0.120 |
| **hopscotch** | infection | | | | 0.349 | 0.558 | | | 4.538 | **0.040** |
|  | colony | | | | 1.485 | 0.235 | | | 15.81 | *<***0.001** |
|  | infection x colony | | | | 2.838 | 0.052 | | | 1.064 | 0.377 |
| **abaecin** | infection | | | | 1.962 | 0.170 | | | 14.73 | *<***0.001** |
|  | colony | | | | 0.924 | 0.439 | | | 8.674 | *<***0.001** |
|  | infection x colony | | | | 1.677 | 0.189 | | | 0.390 | 0.761 |
| **apidaecin** | infection | | | | 0.030 | 0.863 | | | 6.46 | **0.016** |
|  | colony | | | | 2.970 | **0.045** | | | 6.580 | **0.001** |
|  | infection x colony | | | | 1.540 | 0.221 | | | 0.962 | 0.421 |
| **defensin** | infection | | | | 0.316 | 0.578 | | | 0.476 | 0.495 |
|  | colony | | | | 1.389 | 0.262 | | | 2.443 | 0.080 |
|  | infection x colony | | | | 0.476 | 0.701 | | | 0.149 | 0.930 |
| **hymenoptaecin** | infection | | | | 0.017 | 0.898 | | | 0.155 | 0.696 |
|  | colony | | | | 2.295 | 0.094 | | | 0.936 | 0.434 |
|  | infection x colony | | | | 0.822 | 0.490 | | | 2.639 | 0.065 |
| **TEPA** | infection | | | | 0.006 | 0.939 | | | 1.875 | 0.180 |
|  | colony | | | | 0.462 | 0.710 | | | 4.503 | **0.009** |
|  | infection x colony | | | | 2.234 | 0.101 | | | 0.715 | 0.550 |
| **lysozyme 3** | infection | | | | 0.008 | 0.929 | | | 4.241 | **0.047** |
|  | colony | | | | 1.470 | 0.239 | | | 4.697 | **0.007** |
|  | infection x colony | | | | 2.573 | 0.069 | | | 0.640 | 0.595 |
| **transferrin** | infection | | | | 1.885 | 0.178 | | | 1.232 | 0.275 |
|  | colony | | | | 2.084 | 0.120 | | | 10.17 | *<***0.001** |
|  | infection x colony | | | | 0.714 | 0.550 | | | 0.298 | 0.826 |
| **ferritin** | infection | | | | 0.002 | 0.965 | | | 0.005 | 0.944 |
|  | colony | | | | 3.472 | **0.026** | | | 5.959 | **0.002** |
|  | infection x colony | | | | 0.260 | 0.853 | | | 0.595 | 0.623 |
| **jafrac** | infection | | | | 0.489 | 0.489 | | | 0.942 | 0.338 |
|  | colony | | | | 3.751 | **0.019** | | | 3.575 | **0.023** |
|  | infection x colony | | | | 0.910 | 0.446 | | | 0.222 | 0.880 |
| **thioredoxin** | infection | | | | 2.641 | 0.113 | | | 0.020 | 0.887 |
| **dependent** | colony | | | | 3.254 | **0.033** | | | 2.869 | 0.050 |
| **peroxide**  **reductase** | infection x colony | | | | 2.195 | 0.105 | | | 1.438 | 0.248 |
| **peroxiredoxin5** | infection | | | | 0.078 | 0.782 | | | 3.275 | 0.079 |
|  | colony | | | | 5.663 | **0.003** | | | 1.777 | 0.170 |
|  | infection x colony | | | | 1.797 | 0.165 | | | 0.658 | 0.584 |
| **glutathione** | infection | | | | 0.232 | 0.633 | | | 0.492 | 0.488 |
| **S-transferase** | colony | | | | 1.068 | 0.375 | | | 3.420 | **0.028** |
|  | infection x colony | | | | 1.562 | 0.215 | | | 0.107 | 0.956 |
| **Dscam** | infection | | | | 0.075 | 0.786 | | | 1.438 | 0.239 |
|  | colony | | | | 4.599 | **0.008** | | | 1.473 | 0.239 |
|  | infection x colony | | | | 1.101 | 0.361 | | | 1.123 | 0.353 |
| **argonaute** | infection | | | | 0.005 | 0.945 | | | 0.438 | 0.512 |
|  | colony | | | | 0.500 | 0.685 | | | 10.16 | *<***0.001** |
|  | infection x colony | | | | 2.889 | **0.049** | | | 0.285 | 0.836 |
| **aubergine** | infection | | | | 0.154 | 0.697 | | | 0.264 | 0.610 |
|  | colony | | | | 2.335 | 0.090 | | | 3.102 | **0.039** |
|  | infection x colony | | | | 0.975 | 0.415 | | | 0.170 | 0.916 |
| **serpin27a** | infection | | | | 0.165 | 0.687 | | | 4.814 | **0.035** |
|  | colony | | | | 6.382 | **0.001** | | | 2.671 | 0.062 |
|  | infection x colony | | | | 0.652 | 0.587 | | | 0.913 | 0.445 |
| **catsup** | infection | | | | 0.433 | 0.515 | | | 3.669 | 0.064 |
|  | colony | | | | 4.030 | **0.014** | | | 6.046 | **0.002** |
|  | infection x colony | | | | 1.649 | 0.195 | | | 0.709 | 0.553 |
| **punch** | infection | | | | 0.265 | 0.610 | | | 0.475 | 0.495 |
|  | colony | | | | 2.352 | 0.088 | | | 9.495 | *<***0.001** |
|  | infection x colony | | | | 0.766 | 0.521 | | | 0.286 | 0.836 |
| **vitellogenin** | infection | | | | 0.207 | 0.652 | | | 0.519 | 0.476 |
|  | colony | | | | 4.502 | **0.009** | | | 7.836 | *<***0.001** |
|  | infection x colony | | | | 3.262 | **0.032** | | | 0.416 | 0.742 |
| Effects significant at *α* = 5% are highlighted in bold | | | | | | | | | | |

**Figure S1** Gene expression changes upon infection within data subsets for collection sites. Presented values are calculated with the 2^-ddCt^ method. This method yields fold-change values for gene expression between defined sample groups. Error bars are standard errors calculated upon averaging dCt values within sample groups and transformed to fold change errors with error propagation. The solid line marks the value 1 and corresponds to no change between groups. Dashed lines mark the values 2 and 0.5, corresponding to doubled and halved gene expression upon treatment, respectively. Asterisks mark significance of effects as detectable in the univariate outputs of the site specific MANOVAs (Table S4).

Table S7 Description of Linear Discriminant Analyses: R Code (using the MASS package) and full set of LDA coefficients

| **Grouping factor** | **R code** | | | | | | |
| --- | --- | --- | --- | --- | --- | --- | --- |
| site | lda.site <- lda(site~.,data=trans.dCt, prior=c(1,1)/2,na.action="na.omit")  lda.site.jacknified.prediction <- lda(site~.,data=trans.dCt, prior=c(1,1)/2,na.action="na.omit", CV=T) | | | | | | |
| **gene** | **LDA coefficient** | | | | | | |
| PGRP-S3 | 0.545 | | | | | | |
| PGRP-LC | 0.462 | | | | | | |
| BGRP1 | 1.488 | | | | | | |
| BGRP2 | -0.442 | | | | | | |
| hemomucin | -0.226 | | | | | | |
| pelle | 0.838 | | | | | | |
| relish | 0.711 | | | | | | |
| basket | -0.693 | | | | | | |
| hopscotch | -2.643 | | | | | | |
| abaecin | -0.172 | | | | | | |
| apidaecin | 0.042 | | | | | | |
| defensin | -0.242 | | | | | | |
| Hymenoptaecin | 0.239 | | | | | | |
| TEPA | 0.746 | | | | | | |
| lysozyme3 | -0.392 | | | | | | |
| transferrin | -0.701 | | | | | | |
| ferritin | -2.021 | | | | | | |
| jafrac | -0.434 | | | | | | |
| thioredoxin-dependent peroxide reductase | -0.200 | | | | | | |
| peroxiredoxin5 | 4.969 | | | | | | |
| glutathione S-transferase | -0.335 | | | | | | |
| dscam | 0.017 | | | | | | |
| argonaute | 0.461 | | | | | | |
| aubergine | -0.047 | | | | | | |
| serpin27a | -0.813 | | | | | | |
| catsup | 0.458 | | | | | | |
| punch | 0.410 | | | | | | |
| vitellogenin | 0.021 | | | | | | |
|  |  | | | | | | |
| **Grouping factor** | **R code** | | | | | | |
| infection | lda.inf <- lda(infection~., data=trans.dCt, prior=c(1,1)/2, na.action="na.omit")  lda.inf.jacknified.prediction <- lda(infection~.,data=trans.dCt, prior=c(1,1)/2,na.action="na.omit", CV=T) | | | | | | |
| **gene** | **LDA coefficient** | | | | | | |
| PGRP-S3 | 0.378 | | | | | | |
| PGRP-LC | -3.761 | | | | | | |
| BGRP1 | -0.960 | | | | | | |
| BGRP2 | 0.494 | | | | | | |
| hemomucin | 0.919 | | | | | | |
| pelle | 1.470 | | | | | | |
| relish | 1.340 | | | | | | |
| basket | 1.012 | | | | | | |
| hopscotch | -2.400 | | | | | | |
| abaecin | -1.732 | | | | | | |
| apidaecin | 0.612 | | | | | | |
| defensin | 0.303 | | | | | | |
| Hymenoptaecin | 0.233 | | | | | | |
| TEPA | 0.168 | | | | | | |
| lysozyme3 | 0.359 | | | | | | |
| transferrin | 0.206 | | | | | | |
| ferritin | -0.517 | | | | | | |
| jafrac | 1.541 | | | | | | |
| thioredoxin-dependent peroxide reductase | -0.187 | | | | | | |
| peroxiredoxin5 | -1.439 | | | | | | |
| glutathione S-transferase | 0.320 | | | | | | |
| dscam | -0.025 | | | | | | |
| argonaute | 0.0106 | | | | | | |
| aubergine | -0.1506 | | | | | | |
| serpin27a | 0.5816 | | | | | | |
| catsup | -0.0726 | | | | | | |
| punch | 0.746 | | | | | | |
| vitellogenin | 0.204 | | | | | | |
|  |  | | | | | | |
| **Grouping factor** | **R code** | | | | | | |
| colony | lda.col <- lda(colony~.,data=trans.dCt, prior=c(1,1,1,1,1,1,1,1)/8,na.action="na.omit")  lda.col.jacknified.prediction <- lda(colony~.,data=trans.dCt, prior=c(1,1,1,1,1,1,1,1)/8,na.action="na.omit", CV=T) | | | | | | |
| **gene** | **LDA coefficient** | | | | | | |
|  | **LD1** | **LD2** | **LD3** | **LD4** | **LD5** | **LD6** | **LD7** |
| PGRP-S3 | -1.945 | -3.762 | -2.866 | -1.585 | 4.151 | -1.491 | 0.190 |
| PGRP-LC | 1.666 | -0.588 | -0.464 | -2.873 | -1.790 | -0.945 | -0.837 |
| BGRP1 | 0.046 | 0.288 | 1.013 | -0.563 | 0.347 | -0.724 | 0.995 |
| BGRP2 | 0.338 | 1.520 | -0.231 | 1.731 | 1.223 | -0.414 | -0.609 |
| hemomucin | -0.677 | -1.735 | 1.631 | 0.973 | 1.276 | 0.897 | 0.408 |
| pelle | -0.433 | 0.676 | -0.838 | 0.639 | 1.562 | -1.039 | 1.134 |
| relish | -1.472 | 1.060 | -0.105 | -0.023 | 0.428 | 0.219 | 0.303 |
| basket | 2.620 | 2.605 | 2.072 | -0.356 | -4.495 | 0.880 | -1.533 |
| hopscotch | 2.148 | -2.001 | -3.011 | -0.476 | 0.713 | 2.207 | 0.481 |
| abaecin | 0.229 | -1.436 | -0.558 | 0.081 | 0.734 | -0.765 | 0.164 |
| apidaecin | -0.210 | 0.789 | 0.071 | 0.696 | -0.303 | 0.091 | 0.438 |
| defensin | -0.025 | -0.087 | 0.224 | 0.196 | -0.392 | -0.133 | -0.530 |
| hymenoptaecin | -0.237 | 0.147 | 0.016 | -0.222 | 0.238 | 0.119 | 0.109 |
| TEPA | -0.397 | -0.895 | 0.164 | -0.686 | 1.791 | -0.103 | 0.441 |
| lysozyme3 | -0.268 | -0.759 | -0.364 | 0.594 | -0.287 | -0.001 | 0.558 |
| transferrin | 1.955 | 0.735 | 0.478 | -0.032 | -0.737 | 0.735 | -0.444 |
| ferritin | 0.778 | -1.406 | -0.842 | 0.606 | 0.077 | 1.015 | -1.002 |
| jafrac | 2.185 | 3.195 | -0.955 | 0.289 | -2.329 | 1.154 | 1.499 |
| thioredoxin-dependent peroxide reductase | 0.121 | -0.367 | 0.351 | 1.016 | 0.491 | -0.474 | 0.005 |
| peroxiredoxin5 | -2.187 | 4.055 | 5.384 | -2.748 | 1.783 | 0.438 | -0.211 |
| glutathione S-transferase | 0.045 | -0.091 | -0.141 | 0.708 | 0.071 | -0.098 | 0.101 |
| dscam | -0.032 | 0.049 | -0.244 | -0.067 | -0.038 | -0.096 | 0.040 |
| argonaute | 0.410 | 0.074 | 0.346 | -0.322 | -1.875 | -1.246 | -0.012 |
| aubergine | -0.270 | 0.355 | 0.264 | -0.305 | -0.238 | 0.752 | -0.201 |
| serpin27a | 0.362 | 0.716 | -0.065 | 0.613 | -0.793 | 0.477 | -0.666 |
| catsup | 0.183 | -0.036 | 1.231 | -0.281 | 0.281 | -0.471 | -0.846 |
| punch | -1.420 | -0.100 | 0.599 | 0.067 | -0.108 | 0.431 | 0.298 |
| vitellogenin | -0.145 | 0.146 | 0.158 | 0.314 | -0.581 | -0.331 | -0.044 |

**References**

Erler S., Popp M. & Lattorff H.M.G. (2011). Dynamics of immune system gene expression upon bacterial challenge and wounding in a social insect (*Bombus terrestris*). *Plos One*, 6.

Li J.L., Huang J.X., Cai W.Z., Zhao Z.W., Peng W.J. & Wu J. (2010). The vitellogenin of the bumblebee, *Bombus hypocrita*: studies on structural analysis of the cDNA and expression of the mRNA. *Journal of Comparative Physiology B-Biochemical Systemic and Environmental Physiology*, 180, 161-170.

Ramakers C., Ruijter J.M., Deprez R.H.L. & Moorman A.F.M. (2003). Assumption-free analysis of quantitative real-time polymerase chain reaction (PCR) data. *Neuroscience Letters*, 339, 62-66.

Schlüns H., Sadd B.M., Schmid-Hempel P. & Crozier R.H. (2010). Infection with the trypanosome *Crithidia bombi* and expression of immune-related genes in the bumblebee *Bombus terrestris*. *Developmental and Comparative Immunology*, 34, 705 -709.

Vandesompele J., De Preter K., Pattyn F., Poppe B., Van Roy N., De Paepe A. *et al*

(2002). Accurate normalization of real-time quantitative RT-PCR data by

geometric averaging of multiple internal control genes. *Genome Biol*, 3(7)
